# Racial and Ethnic Disparities in Influenza Vaccination among Adults with Chronic Medical Conditions Vary by Age in the United States

**DOI:** 10.1371/journal.pone.0169679

**Published:** 2017-01-12

**Authors:** Degan Lu, Yanru Qiao, Natalie E. Brown, Junling Wang

**Affiliations:** 1 Department of Respiratory Medicine, Shandong Provincial Qianfoshan Hospital, Shandong University, Jinan, Shangdong, China; 2 College of Pharmacy, University of Tennessee, Memphis, Tennessee, United States of America; 3 College of Pharmacy, University of Tennessee Health Science Center, Memphis, Tennessee, United States of America; University of Hong Kong, HONG KONG

## Abstract

**Background:**

People living with chronic health conditions exhibit higher risk for developing severe complications from influenza according to the Centers for Diseases Control and Prevention. Although racial and ethnic disparities in influenza vaccination have been documented, it has not been comprehensively determined whether similar disparities are present among the adult population with at least one such condition.

**Objective:**

To study if racial and ethnic disparities in relation to influenza vaccination are present in adults suffering from at least one chronic condition and if such inequalities differ between age groups.

**Methods:**

The Medical Expenditure Panel Survey (2011–2012) was used to study the adult population (age ≥18) who had at least one chronic health condition. Baseline differences in population traits across racial and ethnic groups were identified using a chi-square test. This was conducted among various age groups. In addition, survey logistic regression was utilized to produce odds ratios of receiving influenza vaccination annually between racial and ethnic groups.

**Results:**

The total sample consisted of 15,499 adults living with at least one chronic health condition. The numbers of non-Hispanic whites (whites), non-Hispanic blacks (blacks), and Hispanics were 8,658, 3,585, and 3,256, respectively. Whites (59.93%) were found to have a higher likelihood of self-reporting their receipt of the influenza vaccine in comparison to the black (48.54%) and Hispanic (48.65%) groups (*P*<0.001). When examining persons aged 50–64 years and ≥65 years, it was noted that the black (54.99%, 62.72%) and Hispanic (53.54%, 64.48%) population had lower rates of influenza vaccine coverage than the white population (59.22%, 77.89) (both *P*<0.0001). No significant differences between whites and the blacks or Hispanics were found among the groups among adults between 18 and 49 inclusive (*P*>0.05). After controlling for patient characteristics, the difference in influenza vaccine coverage between whites and the minority groups were no longer significant for adults aged 50–64 years. However, the difference were still statistically significant for those aged ≥65 years.

**Conclusions:**

In the United States, there are significant disparities in influenza vaccination by race and ethnicity for adults over 65 years with at least one chronic health condition. Future research is needed to help develop more targeted interventions to address these issues and improve influenza vaccination rates.

## Introduction

As a serious public health issue for the Unites States, influenza has significant impact on the utilization and costs of health care. When studying causes of hospitalization during 1979–2001, about 226,000 were found to be due to influenza [1]. Approximately 36,000 deaths occurred annually during 1990–1999 because of influenza and its complications [2]. In 2003, it was estimated that a total of $10.4 billion is spent annually to cover direct medical costs resulting from influenza. The total economic burden due to influenza was reported to be around $87.1 billion in the same year [3].

Effective primary prevention against contracting influenza includes yearly vaccination [4]. As stated by The Advisory Committee on Immunization Practices of the Centers for Disease Control and Prevention, all persons 6 months and older are recommended to receive the influenza vaccination each year [5]. Influenza vaccination in high-risk individuals has also been linked to favorable health outcomes, including fewer illness-related hospitalizations and deaths. Those to be included in the high-risk group are as follows: children aged between 6 and 59 months, adults aged over 49, health-care workers, pregnant women, and those living with at least one chronic health condition [6].

Although universal vaccination against influenza is recommended, population coverage rates (especially for minorities) remain below national goals [7]. Data show that there are disparities in influenza vaccination across racial and ethnic groups [8–10]. Non-Hispanic blacks (blacks) and Hispanics are less likely to receive the immunization in comparison to non-Hispanic whites (whites) [11,12]. These disparities are also experienced among high-risk individuals including the aged population, health-care workers, pregnant women, and individuals with chronic health conditions [13–16].

Because individuals living with at least one chronic health condition have increased susceptibility to developing grave complications from influenza, it is vital for estimations of influenza vaccination disparities to be accurate. Additionally, public health policy can begin to address such disparities when guided by accurate estimates of such disparities. Among existing published studies comparing influenza vaccination coverage across racial and ethnic groups, findings have limited generalizability due to inclusion of only a small subset of chronic diseases [7,12,17–21]. This study aims to (1) evaluate influenza vaccination rates for adults living with at least one chronic condition, and (2) examine racial and ethnic disparities among this population.

## Materials and Methods

### Data source and study population

Data from the 2011–2012 Medical Expenditure Panel Survey (MEPS) were used for this study. Conducted by the Agency for Healthcare Research and Quality (AHRQ), MEPS surveys annual health service usage by non-institutionalized US residents and any related costs, payment sources, and insurance coverage [22]. It is of note that minority populations including blacks and Hispanics were oversampled so more reliable minority population estimates could be made. While MEPS is comprised of several different files, this study used information from both the Full Year Consolidated Data File and the Medical Conditions File. The Full Year Consolidated Data File includes information on respondents’ socio-demographic characteristics, health care usage/cost, and preventative care measures (e.g. influenza vaccination), while the Medical Conditions File catalogs the various health conditions self-reported by survey respondents. The Medical Conditions File also provides information on the International Classification of Diseases, 9th Edition, Clinical Modification (ICD-9-CM) codes and clinical classification codes for disease categories for reported chronic conditions [23].

### Definition of high-risk population

Centers for Disease Control and Prevention (CDC) define persons fitting ≥ one of the following categories as high-risk [6]. The chronic conditions among the following categories were identified based on ICD-9-CM condition codes and clinical classification codes included in the Medical Conditions File in this study. These conditions are self-reported and people with these conditions are at heightened risk for influenza-related complications, hospitalizations, and death [24,25]. Note that this high risk population is defined based on a need factor.

1) Persons with BMI ≥40;

2) Adults living with at least one of the following chronic medical conditions: diabetes; infection with human immunodeficiency virus (HIV); Parkinson’s disease; cancer and maintenance chemotherapy; radiotherapy; epilepsy; sickle cell anemia; asthma; multiple sclerosis; heart valve disorders; paralysis; pulmonary heart disease; cardiac arrest and ventricular fibrillation; acute myocardial infarction; cardiac dysrhythmias; cardiac and circulatory congenital anomalies; chronic obstructive pulmonary disease and bronchi stasis; congestive heart failure; alcohol-related disorders; liver diseases; acute cerebrovascular disease; renal diseases (including nephrosis, nephritis, and renal sclerosis); chronic kidney disease; acute and unspecified renal failure, other diseases of the kidneys and ureters; developmental disorders; delirium, dementia, and other amnestic/cognitive disorders; systemic lupus erythematosus and other connective tissue disorders.

### Outcome variable

The outcome variable was defined as a dummy variable for whether an individual received the influenza vaccine within the year prior to the survey. Responses to a survey question in MEPS were used to ascertain influenza vaccination status. When asked about the most recent receipt of a flu shot, individuals who replied “within the past year” were considered current for influenza vaccination and assigned a “1”. In comparison, respondents who chose “within the past 2 years”, “within the past 3 years”, “within the past 5 years”, “more than 5 years,” or “never” were designated as patients who received no vaccination for influenza in the previous year and assigned a “0”.

### Independent variables

This study used Andersen’s Behavioral Model for Health Services Utilization as the conceptual framework, which guided variables to include for regression models on disparities in influenza vaccination across racial and ethnic groups [26]. Predisposing factors, enabling factors, and need factors were included in the regression models. Age, race, ethnicity, gender, marital status, and geographic region of residence were predisposing factors. Additional population characteristics such as education, household income, health insurance, status of employment, and residence within a Metropolitan Statistical Area (MSA) are all considered enabling factors. An individual’s self-perception of their current health status is considered a need factor.

This study included three mutually exclusive groups including whites, blacks, and Hispanics. Additional independent variables included were: age (18–49 years, 50–64 years, ≥65 years), gender (male/female), marital status (married/not married [including divorced, separated, widowed, or never married]), insurance (including any private, public only, or no insurance), family income (poor = <100% of Federal Poverty Line [FPL], near poor = 100% to <125% of FPL, low income = 125% to <200% of FPL, middle income = 200% to 400% of FPL, high income = ≥400% of FPL), highest degree achieved (<high school, high school or General Educational Development [GED], college [including >high school, associate’s degree, college degree and bachelor’s degree], and beyond college [including master’s degree, doctorate, and professional degree]), employment status (employed/not employed), region (Northeast, Midwest, South, West), urban residence (yes or no), and self-perceived health status (excellent, very good, good, fair, poor)

### Statistical analyses

Survey-weighted chi-square tests were used to compare proportions of vaccinated population between whites and minority groups (blacks and Hispanics). A multivariable logistic regression was utilized to control for patient characteristic in the comparison. Odds ratios (OR) produced by the regression analysis denoted the likelihood of an individual receiving an influenza vaccination within the year before the survey. All analyses considered strata, primary sampling units, and sampling weights of the survey structure of MEPS. This study used SAS 9.4 (SAS Institute Inc., Cary, North Carolina) to perform data analyses. The level of statistical significance was set at p = 0.05.

## Results

### Sample characteristics by race and ethnicity

There were 15,499 adults (weighted to 161,782,372) with at least one chronic medical condition in the total studied population. Of those adults, there were 8,658 whites (weighted to 123,944,979 or 76.61%), 3,585 blacks (weighted to 19,426,484 or 12.01%), and 3,256 Hispanics (weighted to 18,410,909 or 11.38%). Table 1 shows the socio-demographic characteristics for the racial and ethnic groups listed. In comparison to whites, both blacks and Hispanics were younger and less likely to be married, have private insurance, make high incomes, or have a high level of education (*P*<0.05). They are also more likely to reside in MSA and belong to better health categories in self-perceived health status (*P* <0.05). While there were no significant difference in gender distribution among white and Hispanics, there were a higher proportion of females among blacks than among whites. No significant differences in employment status were found between whites and minority groups (*P*>0.05)

**Table 1:** Characteristics of study population across racial and ethnic groups (to be continued), N = 8,658.

Variables	Non-Hispanic whites			Non-Hispanic blacks			Hispanics		
	Number	Weighted frequency	Weighted percentage	Number	Weighted frequency	Weighted percentage	Number	Weighted frequency	Weighted percentage
Age (years)									
18–49	2,654	38,143,826	30.77	1,465	8,284,556	42.65	1,611	8,931,314	48.51
50–64	2,804	40,325,634	32.54	1,149	6,334,781	32.61	973	5,497,240	29.86
≥65	3,200	45,475,518	36.69	971	4,807,147	24.75	672	3,982,355	21.63
Gender									
Male	3,788	55,046,355	44.41	1,272	7,411,707	38.15	1,311	8,012,440	43.52
Female	4,870	68,898,624	55.59	2,313	1,2014,778	61.85	1,945	10,398,469	56.48
Marital status									
Married	4,828	71,986,791	58.08	1,131	6,585,763	33.90	1,690	9,033,377	49.07
Not married^$^	3,830	51,958,188	41.92	2,454	12,840,721	66.10	1,566	9,377,532	50.93
Insurance									
Any private	5,732	87,410,872	70.52	1,682	10,192,575	52.47	1,290	8,583,145	46.62
Public only	2,290	28,608,610	23.08	1,514	7,274,727	37.45	1,272	6,693,572	36.36
No insurance	636	7,925,497	6.39	389	1,959,182	10.09	694	3,134,193	17.02
Family income^$^									
Poor	1,223	13,043,339	10.52	1,094	4,779,487	24.60	868	3,785,767	20.56
Near poor	479	5,782,538	4.67	307	1,528,203	7.87	274	1,314,926	7.14
Low income	1,227	16,068,991	12.96	618	3,190,689	16.42	726	3,956,991	21.49
Middle income	2,552	36,056,817	29.09	976	5,691,030	29.30	892	5,556,531	30.18
High income	3,177	52,993,294	42.76	590	4,237,076	21.81	496	3,796,695	20.62
Highest degree									
Less than high school	1,050	12,817,495	10.36	852	3,800,899	19.73	1,426	6,663,821	36.66
GED^*^or high school	2,893	38,399,244	31.05	1,318	6,557,803	34.05	792	4,545,260	25.01
College^$^	3,754	57,511,782	46.50	1,217	7,779,399	40.39	886	6,212,530	34.18
Beyond college^$^	935	14,947,347	12.09	161	1,122,593	5.83	99	754,231	4.15
Employment status									
Employed	4,162	62,311,706	50.40	1,617	9,466,704	48.88	1645	9,731,549	53.00
Not employed	4,477	61,334,038	49.60	1,958	9,900,698	51.12	1603	8,629,451	47.00
Region									
Northeast	1,424	22,966,541	18.53	604	3,272,351	16.84	576	2,926,494	15.89
Midwest	2,407	31,887,882	25.73	627	3,649,695	18.79	315	1,660,360	9.02
South	3,017	45,764,653	36.92	2,072	10,685,121	55.00	1,076	6,315,131	34.30
West	1,810	23,325,903	18.82	282	1,819,317	9.37	1,289	7,508,924	40.79
Urban residence									
Yes	6,902	99,056,177	79.92	3,112	17,420,847	89.68	3,003	16,967,739	92.16
No	1,756	24,888,802	20.08	473	2,005,638	10.32	253	1,443,170	7.84
Self-perceived health status									
Excellent	1,100	16,461,566	13.40	360	2,057,811	10.66	332	2,046,691	11.19
Very good	2,477	37,377,861	30.43	807	4,641,411	24.04	633	3,865,809	21.14
Good	2,860	40,886,970	33.29	1,266	6,846,031	35.46	1,115	6,250,161	34.18
Fair	1,437	1,9169,096	15.61	820	4,248,322	22.00	905	4,720,274	25.82
Poor	697	8,933,970	7.27	305	1,513,829	7.84	250	1,400,698	7.66

^*^ GED, general equivalency diploma.

^$^ Note. Not married included divorced, separated, widowed, and never married.

Family income: poor, less than 100% of poverty line; near poor, less than 125%; low income, 125% to less than 200%; middle income, 200% to less than 400%; high income, more than 400%.

College included beyond high school, associate degree, college degree, and bachelor’s degree.

Beyond college included master’s degree, doctorate, and professional degree.

### Influenza vaccination coverage among study populations by race and ethnicity

The 2011–2012 overall vaccination coverage rate for adults living with at least one chronic condition was 57.29%. In comparison to whites (59.93%), blacks (48.54%) and Hispanics (48.65%) had a lower likelihood of receiving annual influenza vaccination among this subpopulation (*P*<0.001). Coverage was 39.44%, 59.22%, 77.89% among whites, 35.60%, 54.99%, 62.72% among blacks, and 38.71%, 53.54%, 64.48% among Hispanics for persons with at least one chronic medical condition aged 18–49 years, 50–64 years, and 65 years or older respectively (S1 Fig). Vaccination rates for both black and Hispanic adults aged 50–64 years and ≥65 years were also shown to be statistically significantly lower than their white counterparts (*P*<0.01). However, observed coverage differences in adults 18–49 years between whites and minority populations were not significant (*P*>0.05).

### Regression results

The unadjusted OR based on a logistic regression among adults ≥50 years receiving vaccination for influenza can be found in Table 2. When comparing whites with blacks and whites with Hispanics, the differences found among the groups all proved to be statistically significant (*P*<0.05). After adjusting for patient characteristics in adults between 50–64 years old in a multivariate logistic regression, blacks and Hispanics had a similar likelihood of reporting the receipt of the vaccine (OR of blacks = 0.94, 95% confidence interval [CI] = 0.78–1.14; OR of Hispanics = 1.03, 95% CI = 0.81–1.31). There were still racial and ethnic disparities among individuals over 64 years (OR of blacks = 0.52, 95% CI = 0.41–0.64; OR of Hispanics = 0.66, 95% CI = 0.48–0.91).

**Table 2:** Unadjusted and adjusted odds ratios for influenza vaccination coverage among whites, blacks, and Hispanics aged 50–64 and ≥65 years based on logistic regression.

	UnadjustedOR	95% CI	P-value*	Adjusted OR	95% CI*	P-value*
50–64 years						
Non-Hispanic whites	1.00	Reference		1.00	Reference	
Non-Hispanic blacks	0.84	0.71–0.99	0.049	0.94	0.78–1.14	0.507
Hispanics	0.79	0.65–0.97	0.021	1.03	0.81–1.31	0.802
65 years or older						
Non-Hispanic whites	1.00	Reference		1.00	Reference	
Non-Hispanic blacks	0.48	0.39–0.59	<0.001	0.52	0.41–0.64	<0.001
Hispanics	0.52	0.39–0.68	<0.001	0.66	0.48–0.91	0.011

CI: confidence interval.

* *p* values were calculated using the survey logistic regression model.

## Discussions

Using data from the 2011–2012 MEPS, we found that only 57.29% of adults who had at least one chronic health condition reported to have received vaccination against influenza that year. Compared to the 90% goal set by Healthy People 2020, this coverage rate is suboptimal [27]. The racial and ethnic disparities for influenza vaccination were also found to vary among age groups. Adults aged 18–49 years had a <40% vaccination coverage rate, with no statistically significant differences between racial and ethnic groups compared. For adults aged 50–64 years, both black and Hispanics had lower influenza vaccination coverage rates in comparison to whites. The vaccination disparities among that age group could be explained away by differences in population characteristics across racial and ethnic groups. However, vaccination disparities across the racial and ethnic groups were still significant among adults ≥65 years old. Our findings are consistent with previous studies that examined influenza vaccination disparities in the United States as well [7,8,19,20,28].

Explanation for why influenza vaccination disparities exist across racial and ethnic groups among this high risk group is complex and not well understood. There are many potential contributing factors such as differences in socio-demographic characteristics of patients including socioeconomic and health insurance status [15,20,29]. It has been reported previously that patients had a higher likelihood of receiving vaccination if they were the elderly, married, better educated, and reported better health status [14,30–32]. For instance, La Torre and colleagues found a positive correlation between influenza vaccination and age ≥65 years [31]. Lu et al. also showed elderly individuals who were married had increased likelihood of receiving vaccination [14]. Cohen and colleagues reported that patients with higher education levels are more likely to receive influenza vaccination [33]. Chen and colleagues found that some minorities had a higher likelihood of reporting cost and access barriers for vaccination [19]. In this study, influenza vaccination disparities in adults 50–64 years old may be due to the difference in patient characteristics across racial and ethnic group.

The independent variables included based on Andersen’s Behavioral Model for Health Services Utilization in this study cannot fully account for the racial and ethnic differences found among adults aged ≥65 years. There are still significant gaps in coverage when comparing whites with blacks (48% difference) and whites with Hispanics (34% difference). These racial and ethnic disparities may be caused by a multitude of factors including different quality of health care, mistrust of health care system, low health literacy, negative views on preventative care and vaccination, and insufficient exposure to preventative care [7,8,12,19,34–36].

Both blacks and Hispanics demonstrated a more negative mindset towards influenza vaccination in comparison to whites [15,37]. Not only were they more skeptical about the effectiveness of the vaccination, but they were also of the belief that harmful side effects would result if they were to receive the immunization [19,38]. Health care workers who were black and Hispanic were more concerned about the vaccine’s safety and efficacy profile than white health care workers [39]. A portion of the black population was hesitant to receive the vaccine because they did not trust the health care system due to previous poor experience [40]. These are just a few of the many differences that contribute towards the vaccination disparities between the racial and ethnic groups.

Chronic diseases are the leading cause of disability and death in the United States [41]. In 2012, it was estimated that 50% of the adult population had at least one chronic medical condition. The majority of health care dollars are spent on these chronic diseases [42]. In 2010, 86% of health care costs were spent on patients with chronic conditions [41]. Influenza vaccination distribution programs continue to target this subset of people because they are at higher risk for disease. Mullooly and colleagues reported that patients with high risks experienced higher hospitalization rates than those without among patients ≥50 years old [24]. Ison showed that patients with comorbidities who were hospitalized due to influenza had a higher likelihood of developing complications [43]. Additionally, Matias et al. demonstrated that mortality resulting from influenza was highest among older patients and individual with high risk [44]. While important for everyone, annual influenza vaccination is vital in the elderly population as well as adults living with chronic health conditions. It has also been reported that an influenza patient with specific chronic health conditions would experience higher viral loads and slower viral clearance because the host defense is weakened [45,46].

Reducing racial and ethnic disparities in influenza vaccination is important. Influenza vaccination rates reported in this study were below the 90% target goal for Healthy People 2020 in both the minority and white populations studied [47]. It is estimated that if black and Hispanic influenza vaccination rates were equal to that of whites, an estimated 1,330 black deaths and 550 Hispanic deaths could be prevented [47]. If Health People 2020’s vaccination target goal of 90% was achieved among minority groups, an estimated 3,750 lives would be spared annually [47].

Possible strategies for increasing vaccination rates and reducing corresponding racial and ethnic disparities include but are not limited to improving patients’ knowledge, attitudes, beliefs and access to vaccination on the demand side [48], and encouraging providers to recommend influenza vaccination to patients, especially among racial and ethnic minorities, on the supply side [49]. Interventions on the supply side may be more promising because these patients tend to have more interactions with health care providers than healthy adults and minorities are more likely to be vaccinated in office-based setting [48].

## Limitations

There are limitations with the present study. Firstly, because the influenza vaccination coverage rates were based upon surveyed individuals’ self-report, there is a potential for recall bias. However, self-report of influenza vaccination has been confirmed to be reliable [50,51]. Secondly, the chronic medical conditions outlined in this study were defined using information gathered from self-reports so there is potential for bias. However, existing studies have shown that self-reports can be reliable for conditions that are well-defined and easily diagnosed [52,53]. Therefore, the self-reports used to define these chronic conditions are considered reliable in this study. Lastly, MEPS did not survey how individuals felt personally about influenza vaccination. Consequently, this study was not able to take cultural beliefs and attitudes into account. Future studies should study the different viewpoints on influenza vaccination in order to better explain racial and ethnic disparities in influenza vaccination among older adults.

## Conclusion

The study conducted discovered that influenza vaccination coverage for a high-risk population, adults living with at least one chronic medical condition, was significantly lower than the target goal set by Healthy People 2020. Influenza vaccination disparities for racial and ethnic groups varied significantly between age groups. Although patients aged 18–64 years are negligibly affected, these health disparities greatly impact patients aged 65 years or older. Additional studies on the fundamental causes of racial and ethnic disparities should be conducted in an effort to augment vaccination rates and improve health outcomes.

## Supporting Information

S1 Fig Prevalence of influenza vaccination by age across racial and ethnic groups. (TIF)

S1 Data SAS Output. (MHT)
