# Supplementary figures and images for: Racial and Ethnic Disparities in Influenza Vaccination among Adults with Chronic Medical Conditions Vary by Age in the United States
Source: PLoS One. 2017 Jan 12;12(1):e0169679. doi: 10.1371/journal.pone.0169679 (PMC5231366; doi:10.1371/journal.pone.0169679)

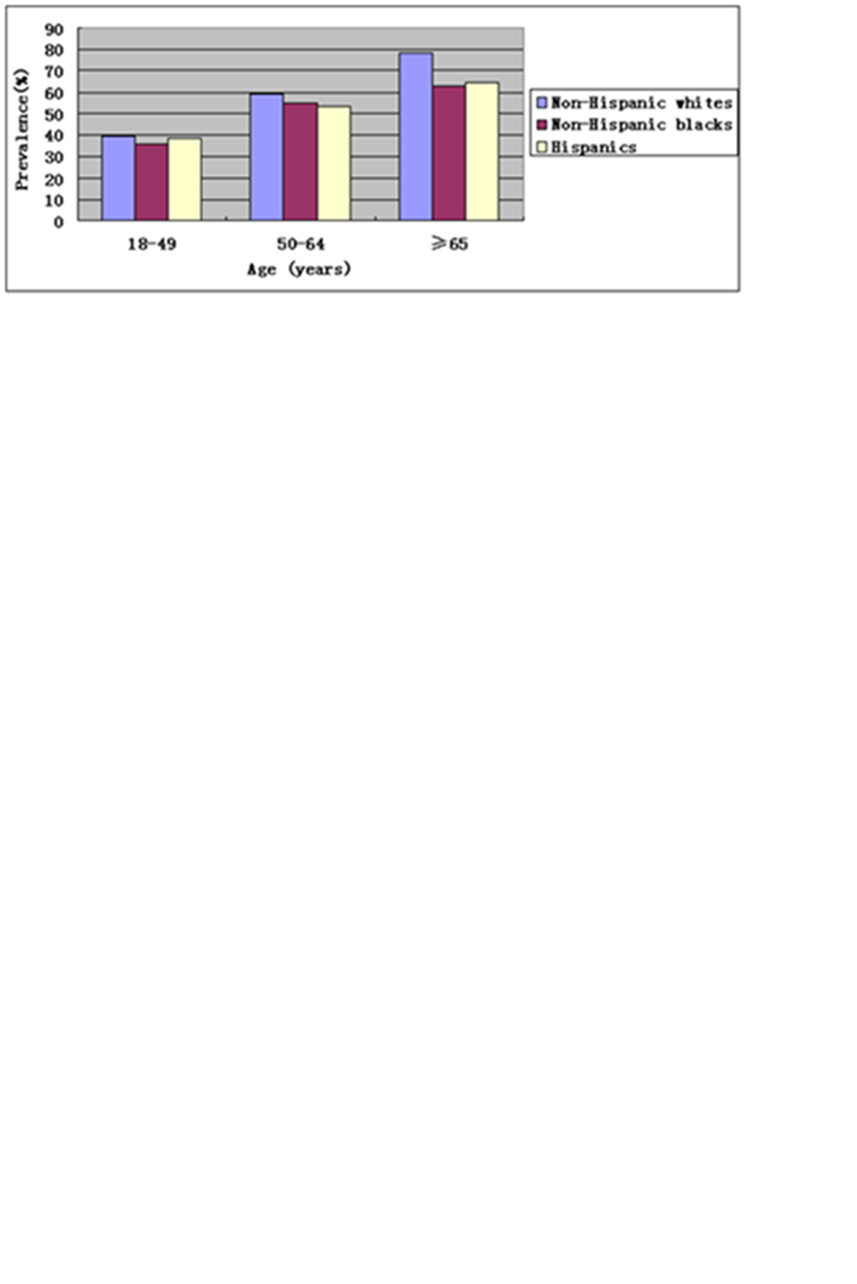

Supplement: S1 Fig — (TIF) [file pone.0169679.s001.tif]
